# On the Potential Energy Surface of the Pyrene Dimer

**DOI:** 10.3390/ijms251910762

**Published:** 2024-10-06

**Authors:** Jiří Czernek, Jiří Brus

**Affiliations:** Institute of Macromolecular Chemistry, Czech Academy of Sciences, Heyrovsky Square 2, 162 00 Prague, Czech Republic; brus@imc.cas.cz

**Keywords:** intermolecular interactions, potential energy surfaces, pyrene dimer, CCSD(T), DLPNO

## Abstract

Knowledge of reliable geometries and associated intermolecular interaction energy (Δ*E*) values at key fragments of the potential energy surface (PES) in the gas phase is indispensable for the modeling of various properties of the pyrene dimer (PYD) and other important aggregate systems of a comparatively large size (ca. 50 atoms). The performance of the domain-based local pair natural orbital (DLPNO) variant of the coupled-cluster theory with singles, doubles and perturbative triples in the complete basis set limit [CCSD(T)/CBS] method for highly accurate predictions of the Δ*E* at a variety of regions of the PES was established for a representative set of pi-stacked dimers, which also includes the PYD. For geometries with the distance between stacked monomers close to a value of such a distance in the Δ*E* minimum structure, an excellent agreement between the canonical CCSD(T)/CBS results and their DLPNO counterparts was found. This finding enabled us to accurately characterize the lowest-lying configurations of the PYD, and the physical origin of their stabilization was thoroughly analyzed. The proposed DLPNO-CCSD(T)/CBS procedure should be applied with the aim of safely locating a global minimum of the PES and firmly establishing the pertaining Δ*E* of even larger dimers in studies of packing motifs of organic electronic devices and other novel materials.

## 1. Introduction

From among the many research directions in the area of intermolecular noncovalent interactions, of particular importance are studies into the structure, stability and electronic properties of dimers of organic aromatic molecules. Their importance is mainly due to the numerous applications of related systems in, for instance, supramolecular junctions [1,2], scaffolds used in the design of organic semi-conductors [3,4] and stimuli-responsive luminescent materials [5,6]. Most recently, a number of joint experimental/theoretical investigations of these dimers appeared, where spectral measurements were accompanied by high-level quantum chemical predictions of the intermolecular interaction energy in the gas phase (abbreviated as Δ*E* in the following) and other properties and where significant structural conclusions were reached (for examples, see references [7,8,9,10] and works cited therein). In this type of study, Δ*E* values were usually obtained from the dispersion-corrected density-functional theory [11] (DC DFT) computations. Notable exceptions are calculations of the Δ*E* performed for pyridine dimers in reference [12] using the explicitly correlated variant [13] of the coupled-cluster theory with singles, doubles and perturbative triples [CCSD(T)] (see the review [14] for background) and for 2-naphthalenethiol dimers in reference [15] using the domain-based local pair natural orbital (DLPNO) approximation [16,17,18] to the CCSD(T). It is noted that currently the “golden standard” Δ*E* predictions require an application of the canonical CCSD(T) computations and extrapolations of pertinent energies to their complete basis set (CBS) limit (see the most recent discussions in references [19,20]), but because of the exceedingly high computational cost of the CCSD(T)/CBS procedure, it cannot be routinely applied yet to larger dimers (with more than about 30 atoms if sufficiently large basis sets were to be used and if the system was of a low symmetry; see reference [21]) to directly support their experimental investigations. It is also noted that the local natural orbital (LNO) CCSD(T) scheme was used to estimate the CBS extrapolated Δ*E* values of systems with more than 100 atoms, but only in their equilibrium geometries [22]. Thus, it should be of interest to obtain the canonical CCSD(T)/CBS Δ*E* results for a mid-sized dimer featuring aromatic stacking, and also in non-equilibrium geometries, to be able to compare these data to their counterparts predicted by some of the reduced-scaling variants of the CCSD(T). This comparison is the first of two main goals of the present paper. In particular, a performance of the focal-point “silver standard” DLPNO-CCSD(T)/CBS approach [23], which involves the iterative treatment of triple excitations within the CCSD(T) [24] together with an application of large basis sets (see Section 4), was inspected in highly complicated bonding scenarios of the cluster containing 52 atoms, namely, the pyrene dimer (PYD). Prior such comparison was carried out in this work, however, the level of the accuracy of the present canonical CCSD(T)/CBS Δ*E* computations themselves was established. This was carried out for ten pi-stacked dimers, each in eight geometries along the dissociation curve, which were taken from the S66x8 set [25] (see Section 2.1). The procedure expressed by Equation (1) in Section 4 was found to be highly accurate and was applied also to a region of the potential energy surface (PES) of the benzene dimer (BD). Namely, 125 structures around the global minimum (GM) of the BD [26] were considered (see Section 2.2). The DLPNO-based method was concomitantly applied to these sets of the S66x8 and BD structures. Based on the quite successful DLPNO-CCSD(T)/CBS results for the challenging smaller systems, the aforementioned comparison with the canonical CCSD(T)/CBS Δ*E* data in an important fragment of the PES was performed for one of the configurations of the PYD, which is a complex frequently studied by calculations and experiments (see references [27,28,29,30,31,32] for the most recent examples, as well as the important solid-state investigation [33] and works cited therein). Specifically, the two sets of CCSD(T)/CBS results were obtained along the dissociation curve of the PYD (see Section 2.3). Importantly, an outstanding level of agreement between the canonical and DLPNO-CCSD(T)/CBS Δ*E* data was found in the region around a minimum of the investigated stacking coordinate. This finding enabled us to attain the second main goal of the present paper, namely, to accurately characterize the Δ*E* minima of the most stable configurations of the PYD (see Section 2.4 for details) and to reveal its GM. The physical origin of the stabilization of these low-lying regions of the PES of the PYD was discussed in Section 3. The DLPNO-CCSD(T)/CBS procedure validated here may be applied in robust searches for the GM of even larger adducts [34].

## 2. Results

### 2.1. Systems from the S66x8 Set

**Figure 1:**
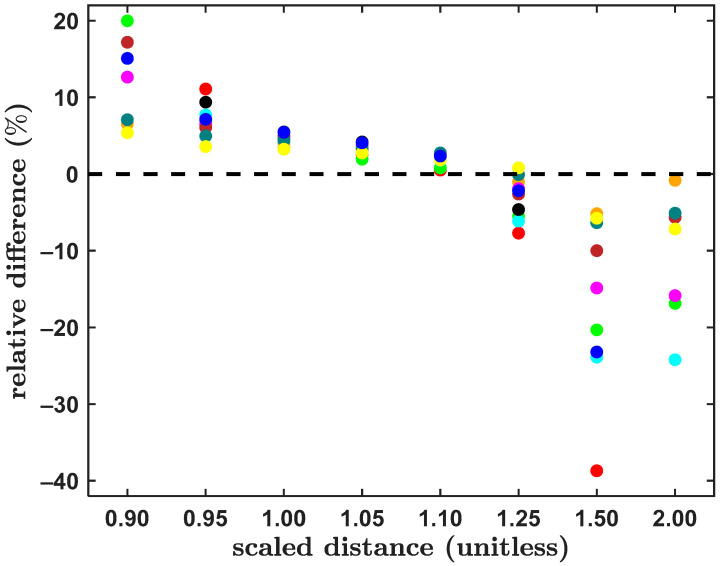
Plot of relative differences between the approximate and canonical interaction energies, which are described in the text, for structures of ten pi-stacked dimers from the S66x8 set [25] in geometries defined by the scaled distance between monomers. Data points are color-coded with the benzene dimer in red, pyridine dimer in green, uracil dimer in orange, benzene–pyridine in cyan, benzene–uracil in magenta, pyridine–uracil in brown, benzene–ethene in black, uracil–ethene in teal, uracil–ethyne in yellow and pyridine–ethene in blue color.

The “golden standard” Δ*E* values from the widely recognized S66x8 benchmark set of small dimers [25] were repeatedly reinvestigated (see references [19,20] and works cited therein). Here, a subset of the S66x8 database was considered. Specifically, all ten pi-stacked systems from the S66x8 set (see Table 1) were used to carefully check the level of the accuracy of the present procedure for obtaining the reference CCSD(T)/CBS Δ*E* data. This procedure is detailed in Section 4. In brief, it applies the pertinent augmented correlation-consistent polarized valence X-tuple basis sets [35,36] (aXZ) to assess contributions to the Δ*E* of Hartree–Fock (HF) energy, the second-order Møller–Plesset correlation energy (MP2) and higher-order correlation energy. In the shorthand notation used in Section 2 and Section 3, these terms are denoted as ${∆E}_{HF}$, ${∆E}_{MP2}$ and ${∆E}_{post-MP2}$, respectively. Of course, a total contribution of the correlation energy, ${∆E}_{corr}$, to the Δ*E* is ${∆E}_{corr}={∆E}_{MP2}+{∆E}_{post-MP2}$, and $∆E={∆E}_{HF}+{∆E}_{corr}$. Importantly, a large aTZ basis set was used to estimate the ${∆E}_{post-MP2}$ term in the focal-point approach (see Equation (1) in Section 4), and the counterpoise correction (CP) [37] was applied throughout. All underlying absolute energies for the Δ*E* estimation using Equation (1) are provided in the Supporting Information in Excel spreadsheets whose names begin with “canonical_”. The CCSD(T)/CBS Δ*E* values obtained in this way for the MP2/cc-pVTZ minima [25] of the aforementioned complexes are listed in Table 1 together with their counterparts from references [19,20] and, wherever available, [22]. Further, a computationally even more demanding procedure was applied to these ten dimers, and its results are also shown in Table 1. Namely, total canonical CCSD(T) energies were obtained while adopting the series of {aDZ; aTZ; aQZ} basis sets, and pertinent values were extrapolated to the CBS limit using the mixed Gaussian/exponential form [38] (see Equation (2) in Section 4) to estimate the Δ*E*. An inspection of Table 1 reveals that the two sets of present results agree very well (within less than 0.2 kJ/mol) with each other, and they also agree with estimates from the literature [19,20,22]. This comparison shows that a very large aQZ basis set is not needed in order to obtain highly accurate CCSD(T)/CBS Δ*E* values. It also shows that the procedure expressed by Equation (1) provides results of benchmark quality. Hence, this procedure was applied also to non-equilibrium geometries of the investigated dimers from the S66x8 set to obtain the reference Δ*E* data. Concomitantly the focal-point method described by Equation (3) of Section 4 was used to approximate the DLPNO-CCSD(T)/CBS Δ*E* values (all pertinent absolute energies can be found in Excel spreadsheets in the Supporting Information). For a total of 80 investigated differences between the DLPNO-CCSD(T)/CBS Δ*E* and their canonical CCSD(T)/CBS counterparts, the mean of absolute values and the mean squared deviation amount to 0.598 and 0.724 kJ/mol, respectively. The highest absolute value of these differences equals ca. 2.8 kJ/mol and is found for the uracil dimer with the relative intermonomer separation, $r_{rel}$, of 0.95$r_{e}$, where $r_{e}$ is the MP2/cc-pVTZ equilibrium distance between monomers [39]. It is stressed that the benchmark value of the Δ*E*, which amounts to –39.162 kJ/mol, agrees well with its “sterling silver” level counterpart of –38.769 kJ/mol from reference [19]. Furthermore, an application of the model expressed by Equation (2) to this highly challenging system [40] yields a very similar result, namely, –39.147 kJ/mol. Relative to these data, the DLPNO-CCSD(T)/CBS value of ca. –36.8 kJ/mol is overestimated by about 7%. Nonetheless, the differences of the DLPNO and canonical CCSD(T) results exhibit a practically uniform (and very interesting) pattern. This is apparent from an inspection of Figure 1, where the relative differences of the two data sets are plotted (it has to be mentioned that seven points with underlying $\left|∆E\right|$ smaller than 1.0 kJ/mol were excluded from analysis so that a division of very small numbers was avoided). In brief, the DLPNO-CCSD(T)/CBS results are systematically underestimated regarding their absolute values for short intermonomer separations, namely, in structures with $r_{rel}$ = 0.90$r_{e}$ and 0.95$r_{e}$, and overestimated for large intermonomer separations, that is, for dimers with $r_{rel}$ = 1.50$r_{e}$ and 2.00$r_{e}$, while there is a quite high level of agreement between the two sets of CCSD(T)/CBS values in the intermediate region, both in absolute and relative terms. The relative differences become the smallest for structures with $r_{rel}$ = 1.10$r_{e}$ (see Figure 1), with a mean value of 2.0%. Regarding the absolute differences obtained for these geometries, their mean absolute value and the mean squared deviation is as low as 0.334 and 0.263 kJ/mol, respectively. This is important because the minima of the CCSD(T)/CBS dissociation curves would be often located in between $r_{rel}$ of 1.05$r_{e}$ and 1.10$r_{e}$ (see Table 1 in reference [19]). As a consequence, the DLPNO approximation to the CCSD(T) can be expected to work very well at “true” minimal geometries. It is worth noting that that mean absolute value of the differences for structures with $r_{rel}$ = 0.90$r_{e}$ and 0.95$r_{e}$ is even higher than 1.0 kJ/mol, amounting to 1.215 and 1.029 kJ/mol, respectively. For these structures, the relative differences can be as high as 20%, which is found for the pyridine dimer with $r_{rel}$ = 0.90$r_{e}$. It is also worth noting that the highest relative differences occur for structures with $r_{rel}$ = 1.50$r_{e}$ (see Figure 1). The biggest value of these differences is found for the pertinent geometry of the benzene dimer and amounts to 38%. In this case the DLPNO-CCSD(T)/CBS computations overestimated the Δ*E* by 0.772 kJ/mol with respect to a rather small reference value of –1.995 kJ/mol. Further results for the BD are presented in the subsequent paragraph.

### 2.2. The Benzene Dimer Structures

**Figure 2:**
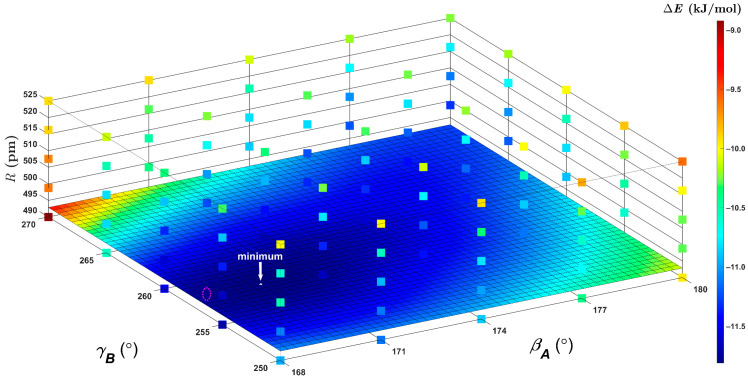
The three-dimensional plot of the intermolecular interaction energies of the benzene dimer that are discussed in the text. The canonical computations place the global minimum with Δ*E* = –11.81 kJ/mol at around R = 493.9 pm, $\beta_{A}$ = 170.0° and $\gamma_{B}$ = 257.4°, as indicated by the white arrow, and the cut through interpolated $∆E(R,\;\beta_{A},\;\gamma_{B})$ data is shown for a value of R in this minimum. The reduced-scaling computations, which were performed on a different grid, predict the global minimum with Δ*E* = –12.15 kJ/mol at around R = 491.3 pm, $\beta_{A}$ = 168.5° and $\gamma_{B}$ = 257.4° (shown schematically as the dotted ellipse in magenta).

**Figure 3:**
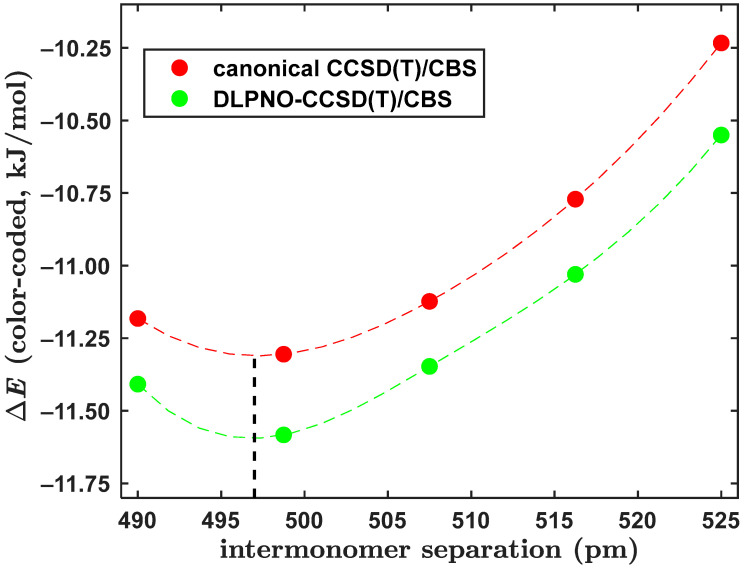
Plot of the distance dependence of the intermolecular interaction energy of the fully symmetric T-shaped dimer of benzene. The respective data points are connected by a spline. An approximate position of the minimum is indicated by the dashed black line drawn at R = 497 pm.

The canonical CCSD(T)/CBS Δ*E* values were predicted using Equation (1) also for an important region of the PES of the BD. The investigated region covered T-shaped structures on the three-dimensional (3D) grid of one radial (R) and two angular ($\beta_{A}$, $\gamma_{B}$) coordinates, which were adopted from reference [41]. Values of these coordinates were chosen in sufficiently wide intervals to contain both the tilted (of C*_s_* symmetry) and fully symmetric (that is, of C_2v_ symmetry) geometries of T-shaped BD structures and to also encompass a position of the GM. Pertinent absolute energies obtained on this 3D grid of 5 × 5 × 5 points are collected in Excel spreadsheets in the Supporting Information together with the Cartesian coordinates of all 125 structures. Figure 2 graphically presents the $∆E(R,\;\beta_{A},\;\gamma_{B})$ data. The canonical CCSD(T)/CBS Δ*E* results span values from a relatively large interval of about 2.80 kJ/mol in a fairly complicated landscape, and their minimum located by 3D interpolation is essentially the same as the one found on a smaller, but tighter, grid in reference [23]. Hence, they served as a stringent test of the DLPNO-CCSD(T)/CBS results obtained by an application of Equation (3). Differences between the two Δ*E* data sets have values between –0.597 and 0.664 kJ/mol, with an average and a median value equal to –0.248 and –0.275 kJ/mol, respectively. The mean squared deviation of these differences is very small, namely, 0.155 kJ/mol. As already mentioned, positions of the GM were obtained by the 3D interpolation of the $∆E(R,\;\beta_{A},\;\gamma_{B})$ data (see Figure 2 for a visualization). Despite the fact that the PES around a position of the GM is very flat [26,41,42,43], an agreement within 3 pm in the radial and 2° in the angular coordinates of the DLPNO and canonical CCSD(T)/CBS minima was found. The corresponding Δ*E* values in these minima differ by about a quarter of kJ/mol only. It is worth noting that for $\beta_{A}$ and $\gamma_{B}$ fixed at 180° and 270°, respectively, the ∆E(R; 180°, 270°) dependence captures a minimum of the fully symmetric geometry of the T-shaped arrangement. The canonical and DLPNO-CCSD(T)/CBS calculations exhibit such a minimum at around the same value of R, namely, R = 497 pm (see Figure 3). Importantly, this value agrees with the result of R = 497.4 pm that was obtained in reference [26] from a robust fit of 19 ∆E(R; 180°, 270°) points predicted by the same canonical CCSD(T)/CBS computational procedure as the one used here. Moreover, the DLPNO-CCSD(T)/CBS Δ*E* data are only slightly and quite uniformly (see Figure 3) overestimated in their absolute value relative to their canonical counterparts. In particular, an average difference between the two sets of Δ*E* values is as low as –0.261 kJ/mol. These results thus confirm an outstanding performance of the present DLPNO-CCSD(T)/CBS approach in regions close to the minima of the PES. In the next part, results for the pyrene dimer are presented.

### 2.3. Dissociation of the Pyrene Dimer

**Figure 4:**
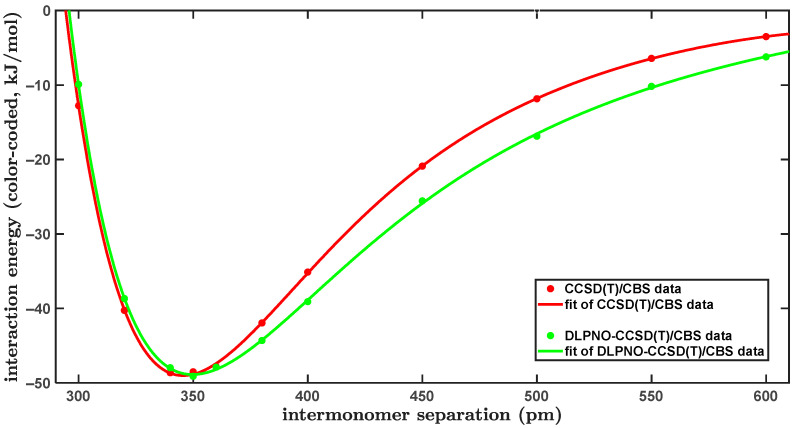
Plot of the distance dependence of the intermolecular interaction energy of the “L” configuration of the pyrene dimer.

The computational models expressed by Equations (1) and (3) were applied to one of the slipped-parallel configurations of the PYD. Here, this topology of the PYD is called “L” in order to use the notation from reference [44] (briefly, the designation “L” refers to a displacement of one of the monomers along the long axis of the reference monomer; see Section 2.4 for further details). The interplane distance, R, was varied in a very wide interval from 300 to 600 pm, and the canonical and DLPNO-approximated CCSD(T)/CBS Δ*E* values were obtained at eleven geometries of this configuration. The geometry of rigid monomers was taken from the Supplementary Information to reference [44]. It is noted that the canonical CCSD(T) calculations were computationally very demanding, as they applied 1932 basis functions of the aTZ basis set to estimate ${∆E}_{post-MP2}$ terms in a fully reliable way [45]. The same form of a modified Dunham expansion (see Section 4) was employed to accurately fit both sets of the CCSD(T)/CBS ∆E(R) data, which are listed in Supporting Information Table S1. Figure 4 graphically presents the results. The ∆E(R) curve fitted to canonical CCSD(T)/CBS values has a minimum of –49.0 kJ/mol at R equal to 346 pm. The corresponding DLPNO-CCSD(T)/CBS ∆E(R) minimum lies very close, namely, at R = 350 pm, and has almost the same Δ*E* value of –48.9 kJ/mol, as might be expected on the basis of results for smaller systems described in preceding paragraphs. Also following these expectations, there is a significant under-binding by the DLPNO-CCSD(T)/CBS computations in the region of short intermolecular distances and a quite strong over-binding for larger R values (see Figure 4). In particular, the canonical CCSD(T)/CBS ∆E(R) curve has an inflexion point at 393 pm with the corresponding Δ*E* of –37.5 kJ/mol. At this intermolecular separation, which can be considered as intermediate, the DLPNO-CCSD(T)/CBS Δ*E* amounts to –40.8 kJ/mol and is thus by about 9% higher in absolute value than its canonical counterpart. Furthermore, the DLPNO-CCSD(T)/CBS ∆E(R) curve has an inflexion point at R = 403 pm with the associated Δ*E* of –38.1 kJ/mol, while the canonical CCSD(T)/CBS Δ*E* result for this distance is lower by ca. 11% in absolute value, as it amounts to –34.2 kJ/mol. These apparent deficiencies of the DLPNO-CCSD(T)/CBS ∆E(R) data are analyzed in Section 3.1 in terms of the respective ${∆E}_{HF}$ and ${∆E}_{corr}$ contributions.

**Figure 5:**
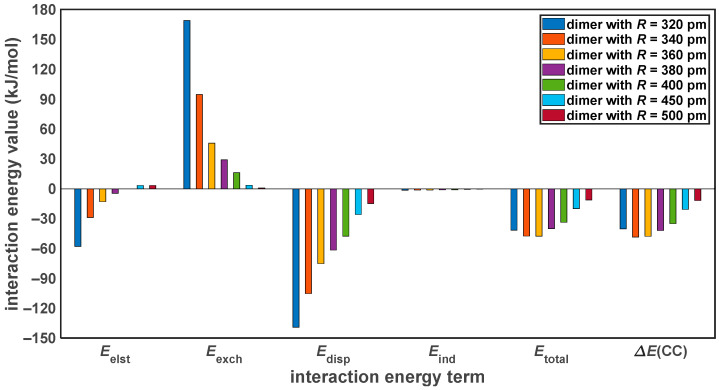
Components of the intermolecular interaction energy at various distances in the “L” configuration of the pyrene dimer.

It is worthwhile to apply the symmetry-adapted perturbation theory (SAPT) of intermolecular interactions [46] and inspect the physical contributions to noncovalent binding in the investigated PYD structures. The SAPT treatment was combined with the DFT-based description of monomers [47] and with extrapolations of all of the SAPT terms to the CBS limit as in our previous work [48] (see Section 4 for technical details and a full set of references). The resulting approach is referred to as SAPT-DFT/CBS and provides reliable values of the electrostatic, $E_{elst}$, Pauli exchange, $E_{exch}$, London dispersion, $E_{disp}$, and induction, $E_{ind}$, contributions to a total intermolecular interaction energy, $E_{total}$, with $E_{total}=E_{elst}+E_{exch}+E_{disp}+E_{ind}$. These contributions are shown in Figure 5 together with the canonical CCSD(T)/CBS interaction energy data, which are denoted as $∆E\left(CC\right)$ to clearly distinguish them from the SAPT result. Within the PYD geometries with the distance R varied in an interval from 320 to 500 pm, the two sets of total interaction energy values agree well. In particular, a value of the mean squared deviation of the seven investigated $\left(E_{total}-∆E\left(CC\right)\right)$ differences equals 1.056 kJ/mol. Significantly, the distance dependence of respective contributions to $E_{total}$ follows the trends typical for systems with aromatic stacking [49,50] and are in accord with results from reference [44]. This SAPT-DFT/CBS approach is thus utilized in the discussion of a stabilization of various PYD configurations in Section 3.2.

### 2.4. Minima of the Pyrene Ddimer

**Figure 6:**
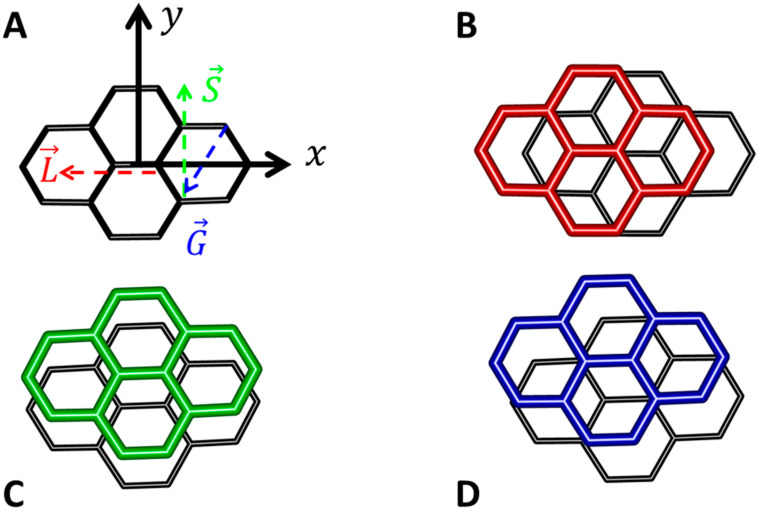
Pictorial representation of the pyrene dimers (all structures are shown to the same scale). (**A**) The reference frame (its *z* axis points towards the viewer and is not shown). (**B**) The slipped-parallel structure of “L” configuration. (**C**) The slipped-parallel structure of “S” configuration. (**D**) The graphene-like structure of “G” configuration.

An outstanding performance of the DLPNO-CCSD(T)/CBS Δ*E* calculations in regions of the PES minima was found for various benchmark sets, as described in previous sections. Consequently, these calculations were used to locate the GM of the PYD by a direct search approach in the rigid-monomer approximation. Due to a relatively high computational cost of some of the underlying computations (in particular, of the iterative CCSD(T) performed with 3704 basis functions of the aQZ basis set), this search was restricted to the lowest-lying fragments of the PES that had been scanned by DFT-based methods in older references [44,51]. The monomer geometry was optimized at the MP2/aTZ level assuming the D_2h_ symmetry. A resulting structure agreed with the experimental geometry of the ground electronic state, that is, *S*_0_ ^1^A_g_ [52]. Specifically, measured values of rotational constants {A, B, C} are {1.001, 0.5593, 0.3610} GHz when rounded, while their MP2/aTZ counterparts accordingly amount to {1.015, 0.5589, 0.3603} GHz. Coordinates of this MP2/aTZ structure are provided in Table S2 together with the atom numbering as employed in setting the angular configurations through translation vectors $\vec{L}$, $\vec{S}$ and $\vec{G}$ (see Figure 6). These vectors are listed in Table S3 and used as in the important study [44] to define the “L”, “S” and “G” topology, respectively, of the PYD. In the coordinate system shown in Figure 6A, the L and S configuration was obtained by offsetting one of the monomers along its long (designated as *x*) and short (*y*) axes, respectively, and keeping the other monomer in its reference geometry. These two parallel-displaced configurations are accordingly pictured in Figure 6B,C. The G (graphite-like) configuration forms a honeycomb pattern, which is shown in Figure 6D. Also investigated here is the “X” (crossed) topology. In this configuration, axes *x* and *y* of one of the monomers are interchanged with respect to the reference orientation, and the resulting dimer resembles a cross. Other structures of the PYD would lie too high on its PES and were not considered as candidates for the GM, while it should be noted that some of the higher-energy structures feature C–H∙∙∙π interactions (see Table III in reference [51]). Thus, the direct search for a minimum of the interplanar distance, R, was carried out for the aforementioned four configurations within their symmetry group, which is specified in Table 2. First the DLPNO-CCSD(T)/CBS Δ*E* values were obtained for distances R spanning an interval from 330 to 380 pm with ten pm step and the resulting ∆E(R) curve was inspected. Then, several additional data points were suitably added in five pm steps for an accurate fit to the functional form given by Equation (4). The final results are presented in Table 2. They indicated the L configuration with the DLPNO-CCSD(T)/CBS optimal R of 343 pm and associated Δ*E* = –52.1 kJ/mol to be the GM, while it should be noted that there were only small differences in the DLPNO-CCSD(T)/CBS ∆E(R) minima of respective configurations, as values of these differences remained within about four kJ/mol and eleven pm for the Δ*E* and R data, respectively (see Table 2). However, the Δ*E* difference between the lowest-lying ∆E(R) minima, namely, of the L and G configurations, was relatively big, amounting to almost two kJ/mol. Moreover, geometries of these two configurations and of an isolated pyrene molecule were fully optimized using the DC DFT method PBE0-D3/TZVPP (see Section 4 for specifications), and the deformation energy of the respective PYD structures was estimated from relevant DLPNO-CCSD(T)/CBS energies. The resulting deformation energy was found to be fairly small, and similar in value, for the L and G configurations (it amounted to 0.396 and 0.462 kJ/mol, respectively). Furthermore, the zero-point energy (ZPE) of these two configurations was estimated through the PBE0-D3/TZVPP harmonic vibrational analysis. A (ZPE(L)–ZPE(G)) difference was found to be 0.316 kJ/mol, which would not change the ordering of the stabilization of the investigated PYD structures. The DLPNO-CCSD(T)/CBS and PBE0-D3/TZVPP-based results thus revealed the L configuration as the GM of the PYD. Interestingly, this configuration was correctly taken as the ground electronic state in the investigation of pyrene excimers, while only the DC DFT calculations of its structure were performed in that work [53] and while two previous DFT-based computations [44,51] both predicted the G configuration to have the highest binding (see also reference [54]). In Section 3.2, a preference for the formation of respective PYD structures is analyzed. By combining the canonical CCSD(T)/CBS Δ*E* of the L configuration (–51.1 kJ/mol), the aforementioned deformation energy of ca. 0.4 kJ/mol and the (ZPE(L)—2 × ZPE(monomer)) difference of 1.6 kJ/mol obtained from the PBE0-D3/TZVP calculations, the dissociation energy of the PYD is predicted to be about –49 kJ/mol.

## 3. Discussion

### 3.1. Discrepancies between the Canonical and DLPNO CCSD(T)/CBS Results

**Figure 7:**
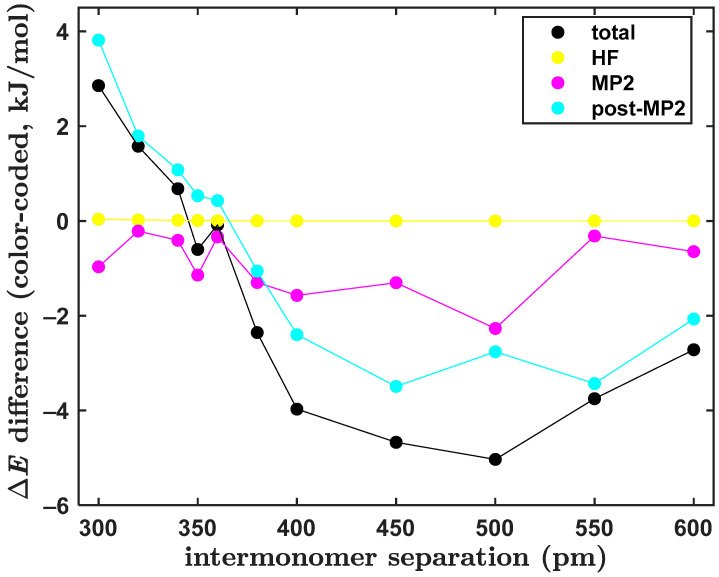
Plot of the distance dependence of the interaction energy differences of the pyrene dimer that are discussed in the text.

Due to their size and complexity, the investigated configurations of the PYD present a challenge to the CCSD(T)/CBS computations. Specifically, a total Δ*E* value of these structures is obtained as a relatively small sum of some large, repulsive ${∆E}_{HF}$, even larger, but attractive ${∆E}_{MP2}$ and a non-negligible, repulsive ${∆E}_{post-MP2}$ components. Hence, it is of interest to inspect the values of these contributions to the ∆E(R) data along the dissociation curve of the PYD since, in Section 2.3, significant differences between the canonical and DLPNO CCSD(T)/CBS Δ*E* results were obtained at small and large interplanar distances R. The underlying data are listed in Table S4. Values of respective components of the Δ*E* vastly vary throughout the investigated range of distances. In particular, the canonical CCSD(T)/CBS ${∆E}_{HF}$, ${∆E}_{MP2}$ and ${∆E}_{post-MP2}$ contributions change by ca. 174, –258 and 74 kJ/mol, respectively, between R = 300 and 600 pm (see Table S4). Figure 7 presents differences of the DLPNO-approximated and canonical CCSD(T)/CBS values of the total ∆E(R) and of its components. A positive value of the differences implies that a weaker binding was predicted by the DLPNO-CCSD(T)/CBS calculation than by its canonical counterpart. Differences of the total interaction energy have values between ca. 2.9 kJ/mol (found at R = 300 pm, which is the shortest distance considered here) and –5.0 kJ/mol (occurs at R = 500 pm). Importantly, these differences are lower than the “spectroscopic accuracy” value of one kJ/mol in an interval from 340 to 360 pm, that is, at around the minima of the investigated ∆E(R) dependences (see Section 2.3, in particular Figure 4). An inspection of Figure 7 reveals that small values of the Δ*E* differences in this region originate from a compensation of errors in the ${∆E}_{MP2}$ and ${∆E}_{post-MP2}$ contributions. Namely, differences of the ${∆E}_{MP2}$ data are negative for all investigated values of R, while differences in the ${∆E}_{post-MP2}$ data are positive from R = 300 up to 360 pm. At the same time, it should be noted that there are essentially no differences between the two sets of the ${∆E}_{HF}$ values throughout the whole range of interplanar distances (see Figure 7). The differences shown in Figure 7 also immediately explain a relatively poor performance of the DLPNO-CCSD(T)/CBS approach in the region of larger values of R. In this part of the ∆E(R) data curves, differences in the ${∆E}_{MP2}$ and ${∆E}_{post-MP2}$ data are of the same sign and both contribute to resulting discrepancies. Specifically, at the ∆E(R) data point with R = 500 pm, the ${∆E}_{MP2}$ and ${∆E}_{post-MP2}$ differences amount to ca. –2.3 and –2.7 kJ/mol, respectively, which leads to the highest absolute value (of 5.0 kJ/mol) of differences between the canonical and DLPNO-CCSD(T)/CBS interaction energies along the investigated curve of the PYD. In this case, also the relative difference is quite high, as it constitutes about 42% of the canonical CCSD(T)/CBS Δ*E* value of ca. –11.8 kJ/mol at R = 500 pm. As a consequence, an application of the DLPNO-CCSD(T)/CBS method, which is computationally quite costly, should be limited to regions lying close to the PES minima in order to obtain the Δ*E* data of benchmark quality. This would likely hold for other “difficult” complexes of a similar and bigger size due to an expected error accumulation of the DLPNO approximation [55].

### 3.2. Stacking Preferences of the Pyrene Dimers

**Figure 8:**
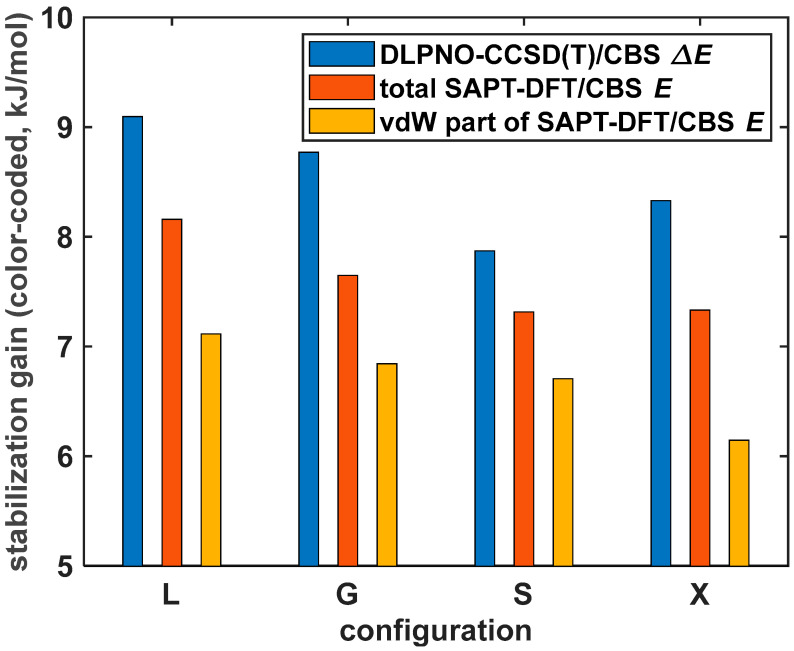
Differences between the interaction energy of {L; G; S; X} configuration and of the fully stacked “sandwich” configuration of the pyrene dimer, which were obtained for the pertinent geometries with the same interplanar separation of R = 370 ppm to describe the stabilization gain of a formation of the investigated structures.

Values of the SAPT-DFT/CBS energy terms are fairly similar for the four PYD configurations described in Section 2.4. This was, of course, expected on the basis of results reported in reference [44]. An illustration of respective contributions to $E_{total}$ data for the set of {L; G; S; X} structures with the same interplanar distances R = 345 and 355 pm is anyway provided in Tables S5 and S6, respectively. The chosen values of R span over a region of the PES minima of the considered configurations (see Table 2). The DLPNO-CCSD(T)/CBS Δ*E* data of these structures are listed in Tables S5 and S6 as well. Importantly, the two sets of total interaction energies have the same ordering, despite a slight overestimation of the binding by the SAPT-DFT/CBS calculations relative to their ab initio counterparts (see Tables S5 and S6). This further confirms, in addition to results from Section 2.3, the reliability of the present SAPT-DFT/CBS computational approach. Hence, it was applied also to the cofacial π∙∙∙π stacked (called “sandwich”) arrangement of the PYD in order to explore a source of the offset stacking of respective {L; G; S; X} structures [56]. The interplanar distance R of 370 pm was chosen for this investigation because it is a value found in the SAPT-DFT/CBS $E_{total}\left(R\right)$ minimum of the sandwich geometries, which were prepared from the same MP2/aTZ monomer as the one used in Section 2.4. The corresponding DLPNO-CCSD(T)/CBS ∆E(R) minimum was located at R = 371 pm, with Δ*E* = –38.2 kJ/mol (see Table 2 for reference). Thus, for the sandwich and {L; G; S; X} structures, all $E_{elst}$, $E_{exch}$, $E_{disp}$ and $E_{ind}$ contributions to $E_{total}$ were inspected in terms of their absolute values and of differences between the sandwich and respective slipped arrangements. Various combinations of the SAPT terms were checked, too, in an attempt to interpret the stacking preferences of the PYD. The “van der Waals” contributions, $E_{vdW}$ [57], $E_{vdW}=E_{exch}+E_{disp}$, turned out to be useful in this respect, contrary to, for instance, sums of the first-order terms of the SAPT expansion ($E_{elst}+E_{exch}$) that were most recently invoked in an analysis of the GM of the benzene dimer [26]. Figure 8 shows various stabilization energies, which were obtained as a difference between the given interaction energy in the sandwich and slipped structures (underlying data are provided in Table S7). Quite similar values of this stabilization were predicted for all four slipped structures. In particular, an average stabilization obtained from the DLPNO-CCSD(T)/CBS Δ*E* and SAPT-DFT/CBS $E_{total}$ results amounted to 8.5 and 7.6 kJ/mol, respectively. The $E_{vdW}$ data captured quite well absolute values of the studied differences (their average is 6.6 kJ/mol). This indicates that π∙∙∙π aromatic interactions are governed by a competition between the $E_{exch}$ and $E_{disp}$ contributions, at least in this part of the PES [58]. However, the ordering of stabilization was swapped by the $E_{vdW}$ results for S and X configurations relative to the DLPNO-CCSD(T)/CBS Δ*E* data, while almost the same values were obtained from SAPT-DFT/CBS $E_{total}$ (see Figure 8 and Table S7).

## 4. Materials and Methods

The canonical CCSD(T)/CBS interaction energy obtained with the CP procedure to reduce the basis set superposition error [37], ${\Delta E}_{CCSD\left(T\right)}^{CBS}$, was predominantly estimated using Equation (1):

(1) $${\Delta E}_{CCSD\left(T\right)}^{CBS}={∆E}_{HF}^{a5Z}+{∆E}_{MP2}^{a5Z}+{∆E}_{post-MP2}^{aTZ}$$

where subscripts denote the respective energy terms, namely, the total Hartree–Fock energy (“HF”), the MP2 correlation energy (“MP2”) and the higher-order correlation energy (“post-MP2”; taken as a difference of the CCSD(T) and MP2 contributions to the total energy [45]), and superscripts specify the augmented correlation-consistent polarized-valenced basis set [35,36], denoted as aXZ, that was used to compute the respective term. In some cases, the ${\Delta E}_{CCSD\left(T\right)}^{CBS}$ value was also established through the model expressed by Equation (2):

(2) $${{\Delta E}_{CCSD\left(T\right)}^{X}=\Delta E}_{CCSD\left(T\right)}^{CBS}+b\exp\left(-\left(x-1\right)\right)+c\exp\left(-{\left(x-1\right)}^{2}\right)$$

where superscript X denotes an application of the aXZ basis set, X∈ (aDZ; aTZ; aQZ), for obtaining the total CCSD(T) energy, while the corresponding integer value of x is 2, 3 and 4, respectively. Thus, three equations in three unknowns (namely, ${\Delta E}_{CCSD\left(T\right)}^{CBS}$, b and c) were formed in this model and solved analytically for a value of ${\Delta E}_{CCSD\left(T\right)}^{CBS}$. Calculations of all the aforementioned CCSD(T) energies were performed in the Molpro version 2022.2 [59]. The HF/a5Z and MP2/a5Z energies for Equation (1) were computed using the Turbomole version 7.1 [60]. The MP2/a5Z correlation energies were obtained in the resolution-of-the-identity integral approximation [61,62] while applying the relevant auxiliary basis sets [62].

The DLPNO-CCSD(T)/CBS interaction energy (denoted simply as Δ*E* below) was estimated using the previously developed procedure [63]. This procedure is described by Equation (3) (the notation as in Equation (1) is used, and the right arrow symbol indicates an application of the two-point extrapolation formula from reference [64]):

(3) $$∆E={∆E}_{HF}^{aQZ}+{∆E}_{MP2}^{aTZ\to aQZ}+{∆E}_{post-MP2}^{aTZ\to aQZ}$$

Of course, the underlying CCSD(T) and MP2 correlation energies were obtained in the DLPNO approximation [16,17,18,65], and the CP was applied. The ORCA version 5.0.3 [66] was used. The HF calculations applied “VeryTightSCF” accuracy settings. The default method of the orbital localization was adopted, and the “T1” option for the iterative treatment of triple excitations within the CCSD(T) method [24] was used. The electron-correlation space was truncated through the “TightPNO” set of parameters.

The density-fitting variant of the SAPT-DFT method [67] was used as implemented in Molpro 2022.2. Previously developed computational protocol [68] was applied in order to estimate the respective SAPT-DFT/CBS terms. In brief, the $E_{elst}$, $E_{exch}$, $E_{disp}$ and $E_{ind}$ contributions to the total interaction energy, $E_{total}$, are related to the underlying interaction energy terms as follows: $E_{elst}$ and $E_{exch}$ are the electrostatic polarization and Pauli exchange energy contributions, respectively, arising in the first order of the perturbation theory of intermolecular interactions [69]; $E_{disp}$ is the London dispersion energy contribution obtained as a sum of the second-order terms $E_{disp.}^{SAPT\;(2)}$ and $E_{disp.-exch.}^{SAPT\;(2)}$ [70]; and $E_{ind}$ is the induction energy contribution approximated by a sum of the second-order terms $E_{ind.}^{SAPT\;(2)}$ and $E_{ind.-exch.}^{SAPT\;(2)}$ [71] and of the correction term $E_{\delta (HF)}^{SAPT}$, which is computed at the HF level [72].

The least-squares fit of pertinent $∆E\left(R\right)$ data employed the following functional form:

(4) $$∆E\left(R;\;r_{e},a_{0},a_{1},a_{2},a_{3},a_{4},a_{5},a_{6},V_{e}\right)=a_{0}\xi^{2}\left(1+a_{1}\xi +a_{2}\xi^{2}+a_{3}\xi^{3}+a_{4}\xi^{4}+a_{5}\xi^{5}+a_{6}\xi^{6}\right)+V_{e}$$

where $\xi =\left(R-r_{e}\right)/R$ and R is the interplanar distance. The trust-region-reflective algorithm from the “lsqcurvefit” function of MATLAB^®^ Optimization Toolbox™ was applied to obtain final sets of $\left(r_{e},a_{0},a_{1},a_{2},a_{3},a_{4},a_{5},a_{6},V_{e}\right)$ results.

The PBE0-D3/def2-TZVPP approach (the PBE0 hybrid-functional [73] applied together with the D3 empirical dispersion correction [74] and the triple zeta valence basis set from reference [75]) was used to optimize the geometries for an assessment of the deformation energy. It was also used to obtain the harmonic vibrational frequencies for an estimation of the ZPE differences. In the underlying calculations, the Gaussian 16 revision C.01 [76] was applied with default algorithms and settings.

## 5. Conclusions

To conclude, this study has achieved two major goals. The first major goal was to obtain fully reliable canonical CCSD(T)/CBS Δ*E* values along the π∙∙∙π stacking coordinate in a large adduct, namely, the PYD containing 52 atoms, for the purpose of their comparison to the “silver standard” DLPNO-CCSD(T)/CBS Δ*E* results. The comparison revealed an excellent agreement of the two sets of Δ*E* data around the $∆E\left(R\right)$ minima. In fact, these minima were found to be almost identical. However, this agreement was shown to be a result of the compensation of errors of the DLPNO-based ${∆E}_{MP2}$ and ${∆E}_{post-MP2}$ contributions to the intermolecular binding relative to their canonical counterparts. Consequently, the canonical CCSD(T)/CBS calculations would be needed for obtaining the benchmark Δ*E* values at non-equilibrium geometries of similarly complex systems. The second major goal was to exploit the aforementioned agreement and use the DLPNO-CCSD(T)/CBS strategy for an accurate characterization of the most stable PYD structures. Namely, four low-lying regions of the PES were investigated, which corresponded to {L; G; S; X} configurations around their minima. From among them, the “L” configuration with the DLPNO-CCSD(T)/CBS optimal stacking distance of 343 pm and associated Δ*E* = –52.1 kJ/mol was found to be the GM. These values are recommended for use in investigations involving the ground electronic state of the PYD [77]. Using the computed data for the GM and an isolated pyrene, the dissociation energy of the PYD in the gas phase was estimated to be about –49 kJ/mol, which awaits experimental verification.

## Figures and Tables

**Table 1:** Comparison of the predicted interaction energy values for minima of ten pi-stacked complexes from the Set66 [25].

System (Designation in S66 Set)	CCSD(T)/CBS |Δ*E*| Estimate/kJ/mol				
	From Equation (1)	From Equation (2)	From Ref. [22] ^(a)^	From Ref. [20] ^(b)^	From Ref. [19] ^(c)^
benzene–benzene (S24)	10.926	10.762	11.171 ± 0.293	11.238	10.548
pyridine–pyridine (S25)	15.462	15.340	15.481 ± 0.335	15.731	15.104
uracil–uracil (S26)	40.578	40.593	40.203 ± 0.418	40.652	40.246
benzene–pyridine (S27)	13.561	13.428	13.724 ± 0.293	13.824	13.205
benzene–uracil (S28)	23.239	23.101	22.928 ± 0.460	23.200	22.866
pyridine–uracil (S29)	27.905	27.774	27.656 ± 0.377	27.870	27.514
benzene–ethene (S30)	5.570	5.482	—	5.619	5.310
uracil–ethene (S31)	13.860	13.800	—	13.823	13.627
uracil–ethyne (S32)	15.362	15.333	—	15.376	15.008
pyridine–ethene (S33)	7.455	7.378	—	7.464	7.201

^(a)^ Obtained using the LNO scheme; ^(b)^ the “14k-GOLD” level result; ^(c)^ the “sterling silver” level result.

**Table 2:** Computational results for the four low-lying configurations of the pyrene dimer.

Parameter	Configuration (Symmetry)			
	L (C_2h_)	G (C_s_)	S (C_2h_)	X (D_2h_)
R/pm	344	347	350	355
Δ*E*/kJ/mol	–52.1	–50.3	–49.1	–48.4

## Data Availability

The data presented in this study are available in the article and in the Supplementary Materials.

## Supplementary Materials

The following supporting information can be downloaded at https://www.mdpi.com/article/10.3390/ijms251910762/s1.
