# Supplementary material for: On the Potential Energy Surface of the Pyrene Dimer
Source: Int J Mol Sci. 2024 Oct 6;25(19):10762. doi: 10.3390/ijms251910762 (PMC11476719; doi:10.3390/ijms251910762)
Supplement: Supplementary file 1 [file ijms-25-10762-s001.zip › SI/SI.pdf]

Supplementary Information to "On the potential energy surface of the pyrene dimer" by Czernek & Brus (*IJMS* **2024**)

Table of Contents

Table S1 at page S2: data for Figure 4;

Table S2 at page S3: the reference MP2/aTZ geometry of pyrene;

Table S3 at page S3: definition of vectors shown in Figure 6;

Table S4 at page S4: values of the interaction energy terms along the dissociation curve;

Table S5 at page S5: values of the interaction energy terms for structures with R = 345 pm;

Table S6 at page S6: values of the interaction energy terms for structures with R = 355 pm;

Table S7 at page S7: values of the interaction energy terms for structures with R = 370 pm.

Table S1. The CCSD(T)/CBS $\Delta E(R)$ data actually used for fitting of the curves that are shown in Figure 4.

R (in pm)	canonical (in kJ/mol)	DLPNO (in kJ/mol)
300.0	–12.774401702968419	–9.919799420317418
320.0	–40.260526630188792	–38.684553755421177
340.0	–48.645685531426068	–47.966382645824808
350.0	–48.512581676332729	–49.115730325036566
360.0	–47.815582042281676	–47.900646583327216
380.0	–41.956491502178437	–44.313717010396545
400.0	–35.118717975617585	–39.091430045555001
450.0	–20.891534155437796	–25.564523303038129
500.0	–11.838733831853517	–16.871666653276009
550.0	–6.423990652332975	–10.176298351478636
600.0	–3.499679191334963	–6.218363133252922

Table S2. The MP2/aTZ coordinates (in Å) of pyrene and numbering of its atoms as used to prepare the PYD structures.

atom	*x*	*y*	*z*
C1	–0.7082270	0.0	0.0
C2	0.7082270	0.0	0.0
C3	1.4220460	1.2339380	0.0
C4	1.4220460	–1.2339380	0.0
C5	–1.4220460	–1.2339380	0.0
C6	–1.4220460	1.2339380	0.0
C7	0.6828120	2.4564060	0.0
C8	2.8235010	1.2096000	0.0
C9	0.6828120	–2.4564060	0.0
C10	2.8235010	–1.2096000	0.0
C11	–0.6828120	–2.4564060	0.0
C12	–2.8235010	–1.2096000	0.0
C13	–0.6828120	2.4564060	0.0
C14	–2.8235010	1.2096000	0.0
C15	3.5134940	0.0	0.0
C16	–3.5134940	0.0	0.0
H17	1.2301430	3.3924680	0.0
H18	3.3671020	2.1475670	0.0
H19	1.2301430	–3.3924680	0.0
H20	3.3671020	–2.1475670	0.0
H21	–1.2301430	–3.3924680	0.0
H22	–3.3671020	–2.1475670	0.0
H23	–1.2301430	3.3924680	0.0
H24	–3.3671020	2.1475670	0.0
H25	4.5962120	0.0	0.0
H26	–4.5962120	0.0	0.0

Table S3. The vectors shown in Figure 6.

configuration	L	G	S
vector	$\vec{L} = \frac{1}{2}(\mathrm{C1} - 6\mathrm{C1})$	$\vec{G} = \frac{1}{2}(\mathrm{C8} - \mathrm{C4})$	$\vec{S} = \frac{1}{2}(\mathrm{C4} - \mathrm{C3})$

Table S4. Values of the interaction energy terms, in kJ/mol, along the dissociation curve of the PYD.

R (in pm)	from Equation 1				from Equation 3			
	ΔE_{HF}	ΔE_{MP2}	$\Delta E_{\mathrm{post-MP2}}$	ΔE	ΔE_{HF}	ΔE_{MP2}	$\Delta E_{\mathrm{post-MP2}}$	ΔE
300.0	176.4862	−265.9536	76.693	−12.7744	176.5205	−266.9484	80.5081	−9.9198
320.0	102.8758	−202.0931	58.9568	−40.2605	102.8974	−202.3328	60.7508	−38.6846
340.0	60.5221	−154.7953	45.6276	−48.6457	60.5338	−155.2047	46.7046	−47.9664
350.0	46.7483	−135.5022	40.2413	−48.5126	46.7561	−136.6456	40.7738	−49.1157
360.0	36.3619	−119.7239	35.5464	−47.8156	36.3666	−120.1285	35.8613	−47.9006
380.0	22.6379	−92.4806	27.8861	−41.9565	22.6389	−93.7808	26.8282	−44.3137
400.0	14.8287	−71.9773	22.0298	−35.1187	14.8284	−73.5488	19.629	−39.0914
450.0	6.8108	−40.294	12.5917	−20.8915	6.8093	−41.4719	9.0981	−25.5645
500.0	4.3799	−23.7198	7.5011	−11.8387	4.3789	−25.9901	4.7395	−16.8717
550.0	3.3036	−14.3642	4.6366	−6.4240	3.3038	−14.683	1.2029	−10.1763
600.0	2.6297	−9.2000	3.0706	−3.4997	2.6298	−9.8483	1.0001	−6.2184

Table S5. The CBS-extrapolated values (in kJ/mol) of the interaction energy terms of the investigated configurations featuring the same interplanar distance R = 345 pm.

Term	Configuration				
	L	G	S	X	sandwich
E_{elst}	–23.5108	–23.6804	–24.0156	–25.6709	–28.8477
E_{exch}	75.6091	78.7840	81.3756	89.6625	109.6854
$E_{\text{ind.}}^{\text{SAPT (2)}}$	–32.5371	–33.5946	–34.3827	–37.3884	–44.1479
$E_{\text{ind.-exch.}}^{\text{SAPT (2)}}$	31.1642	32.2586	33.0879	36.1656	43.3828
$E_{\text{disp.}}^{\text{SAPT (2)}}$	–110.5156	–112.9797	–115.0522	–121.1951	–128.7519
$E_{\text{disp.-exch.}}^{\text{SAPT (2)}}$	15.9262	16.4741	16.8935	18.2577	20.7538
$E_{\delta(\text{HF})}^{\text{SAPT}}$	–5.9903	–5.8561	–5.5032	–6.1611	–1.8221
E_{total}	–49.8544	–48.5940	–47.5967	–46.3296	–29.7475
ΔE	–52.0760	–50.2286	–48.7303	–47.4496	–28.0795

Table S6. The CBS-extrapolated values (in kJ/mol) of the interaction energy terms of the investigated configurations featuring the same interplanar distance R = 355 pm.

Term	Configuration				
	L	G	S	X	sandwich
E_{elst}	–16.0240	–16.0264	–16.2014	–17.1643	–19.1283
E_{exch}	56.2868	58.7095	60.6803	66.7497	81.6309
$E_{\text{ind.}}^{\text{SAPT (2)}}$	–23.5713	–24.3294	–24.8963	–26.9477	–31.6288
$E_{\text{ind.−exch.}}^{\text{SAPT (2)}}$	22.2589	23.0432	23.6409	25.7440	30.7668
$E_{\text{disp.}}^{\text{SAPT (2)}}$	–95.0707	–97.1617	–98.9239	–104.0566	–110.2996
$E_{\text{disp.−exch.}}^{\text{SAPT (2)}}$	12.4539	12.8863	13.2181	14.2677	16.2098
$E_{\delta(\text{HF})}^{\text{SAPT}}$	–4.3732	–4.2624	–3.9895	–4.4690	–1.4401
E_{total}	–48.0396	–47.1409	–46.4718	–45.8762	–33.8894
ΔE	–50.9296	–49.2731	–49.0551	–48.3904	–35.1603

Table S7. The CBS-extrapolated values (in kJ/mol) of the interaction energy terms of the investigated configurations featuring the same interplanar distance R = 370 pm.

Term	Configuration				
	L	G	S	X	sandwich
E_{elst}	–8.3341	–8.1917	–8.1970	–8.5118	–9.3176
E_{exch}	36.0434	37.6666	38.9420	42.7365	52.2410
$E_{\mathrm{ind.}}^{\mathrm{SAPT\,(2)}}$	–14.6130	–15.0760	–15.4062	–16.5593	–19.2425
$E_{\mathrm{ind.-exch.}}^{\mathrm{SAPT\,(2)}}$	13.3908	13.8703	14.2199	15.4045	18.3016
$E_{\mathrm{disp.}}^{\mathrm{SAPT\,(2)}}$	–76.2294	–77.8855	–79.2505	–83.1881	–87.8693
$E_{\mathrm{disp.-exch.}}^{\mathrm{SAPT\,(2)}}$	8.5702	8.8748	9.1007	9.8052	11.1264
$E_{\delta(\mathrm{HF})}^{\mathrm{SAPT}}$	–2.6894	–2.6088	–2.4259	–2.7200	–0.9418
E_{total}	–43.8613	–43.3502	–43.0170	–43.0330	–35.7022
ΔE	–47.3915	–47.0666	–46.1678	–46.6258	–38.2962
